# Therapeutic implications of cellular and molecular biology of cancer stem cells in melanoma

**DOI:** 10.1186/s12943-016-0578-3

**Published:** 2017-01-30

**Authors:** Dhiraj Kumar, Mahadeo Gorain, Gautam Kundu, Gopal C. Kundu

**Affiliations:** 1 Laboratory of Tumor Biology, Angiogenesis and Nanomedicine Research, National Centre for Cell Science (NCCS), Pune, 411007, India; 2 Deapartment of Biology, Northeastern University, Boston, MA 02115, USA

**Keywords:** CSCs, Signaling, Microenvironment, Angiogenesis, Metastasis, Melanoma growth

## Abstract

Melanoma is a form of cancer that initiates in melanocytes. Melanoma has multiple phenotypically distinct subpopulation of cells, some of them have embryonic like plasticity which are involved in self-renewal, tumor initiation, metastasis and progression and provide reservoir of therapeutically resistant cells. Cancer stem cells (CSCs) can be identified and characterized based on various unique cell surface and intracellular markers. CSCs exhibit different molecular pattern with respect to non-CSCs. They maintain their stemness and chemoresistant features through specific signaling cascades. CSCs are weak in immunogenicity and act as immunosupressor in the host system. Melanoma treatment becomes difficult and survival is greatly reduced when the patient develop metastasis. Standard conventional oncology treatments such as chemotherapy, radiotherapy and surgical resection are only responsible for shrinking the bulk of the tumor mass and tumor tends to relapse. Thus, targeting CSCs and their microenvironment niche addresses the alternative of traditional cancer therapy. Combined use of CSCs targeted and traditional therapies may kill the bulk tumor and CSCs and offer a promising therapeutic strategy for the management of melanoma.

## Background

Tumor initiating cells having stem cell characteristics were first discovered in leukaemia and later in solid tumors which recently has become an important area in cancer research [1]. These stem like tumor cells, termed as cancer stem cells (CSCs) govern tumor progression, angiogenesis and metastasis via modulating certain specific pathways which depends upon the type of the tissue. CSCs have similar physiological properties as normal stem cells, like self-renewal, differentiation and indefinite proliferation ability which might be the main cause of tumor progression [1]. Conventional anti-cancer treatments eradicate bulk of tumor mass but it is ineffective for CSCs and hence could be the reason for tumor reoccurrence and progression. CSCs have been identified in hematopoietic cancer and solid tumors like brain, breast, prostrate, colon, pancreatic, lung and most recently in melanoma.

Malignant melanoma is a highly aggressive and drug-resistant cancer [2]. Several groups have shown the existence of tumor heterogeneity with undifferentiated molecular signatures having high tumorigenic potential with embryonic-like differentiation which strongly suggest the presence and the involvement of CSCs in melanoma. Although the concept of CSCs is well accepted for many tumors, but the existence of CSCs in melanoma has been the subject of debate. Initially, Fang et al. and Monzani et al. have shown the existence of stem cell-like subpopulation in CD20^+^ and CD133^+^ melanoma cells [3, 4]. Subsequent studies support the involvement of CSCs in human melanoma progression using ABCB5 and CD271 as markers [5, 6]. Recently, Luo et al. have provided significant evidence and shown the existence of CSCs in melanoma by using ALDH, an intercellular stem cell marker in melanoma [7]. Moreover, CSCs are responsible for EMT, metastasis and angiogenesis in autocrine or paracrine manner [8, 9]. Tumor microenvironment also plays a major role during the melanoma progression. For example, stroma-derived osteopontin regulates the side population (SP) enrichment and controls angiogenesis and metastasis in melanoma [10]. Hypoxia inducible factor (HIF) and transcription factor like Snail are expressed in CSCs derived from glioma and melanoma that leads to enrichment of CSC, self-renewal and differentiation and control angiogenesis and metastasis [11, 12]. CSCs are responsible for recurrence in most of tumor which associated with modulation of tumor microenvironment and immune escape mechanisms [13]. Many studies showed that CSCs exhibit specific intracellular molecular properties that are distinct with their rest of the bulk tumor cells which lead to limited response against conventional treatments [14, 15]. Additionally, the expression of various miRNAs in CSCs strongly correlates with melanoma progression which helps in the modulation of tumor microenvironment through targeting the various specific signaling pathway [16–18]. Traditional chemotherapy or radiation therapies are not sufficient to eliminate CSCs from the tumors, therefore, understanding the cellular and molecular biology of CSCs are essential for the identification of novel CSCs-targeted therapies.

### Melanoma CSCs and their unique markers

Several lines of evidences suggested the presence and involvement of CSCs in melanoma initiation and progression [3]. Identification of highly aggressive undifferentiated subpopulations with embryonic-like plasticity within the melanoma has established the link between the tumor progression and CSCs [3, 4]. The melanoma derived spheres demonstrated a significant differentiation potential capable of giving rise to melanocytes, adipocytes, osteocytes and chondrocytes. These spheres also exhibit high self-renewal ability both in vitro and in vivo [3]. CSCs are thought to express cell surface and intracellular markers traditionally associated with tissue specific stem cells which are responsible for tumor heterogeneity [19]. Earlier studies suggested that melanoma stem cells can be characterized based on the expression of markers such as CD20, CD133 and MDR1 as shown in Table 1. However, a firm correlation between expression of markers with various other properties such as self-renewal ability, high tumorigenic potential, multilineage differentiation in CSCs is yet to be studied [3–5].

**Table 1:** CSCs markers are used for their characterizations in melanoma

Markers	Associated properties and functions	References
CD133	• Tumor initiation • Maintain long-term tumorigenic potential • Chemoresistance • Activates p38 MAPK pathway • Induces metastasis and angiogenesis	[4, 15]
ABCG2	• Tumor initiation	[4]
CD271	• Associated with metastasis • Establish tumor heterogeneity • Maintain long-term tumor growth	[6]
ABCB5	• Self-renewal and differentiation • Tumor initiation • Vasculogenic mimicry	[5, 9]
ALDH	• Self-renewal and differentiation • Highly tumorigenic • Chemoresistance	[7]
CD20	• Highly enrich in melanosphere • Exhibit self-renewal	[3]
PD-1	• Highly tumorigenic • Help in the evasion of tumor immunity	[25]
VEGFR1	• Higher tumor growth • Vasculogenic mimicry • Coexpressed with ABCB5	[9]
CXCR6	• Self-renewal • Highly tumorigenic	[27]
JARID1B	• Give rise to highly proliferative progeny • Self-renewal • Exhibits continuous tumor growth and metastasis • Regulates Jagged1/Notch1 signaling	[30]

Previous studies demonstrate that melanoma cells or clinical specimens undergoing chemoresistance overexpress a number of stem cell markers including CD133 and ABCG2 [4]. Furthermore, Nordvig et al. reported that CD133^+^ keratinocytes exhibit high mitochondrial potential that may have clinical implications in non-melanoma skin cancer [20]. Roudi et al. have studied gene-expression profiling in CD133^+^ cells compared with CD133^−^ D10 cells. The data demonstrated that 130 genes were upregulated including ABC transporter super-family (ABCC1, ABCG2 and ABCC6), while 61 genes were downregulated including apoptosis modifying genes (CASP8 and TNFRSF4). These data indicate that CD133^+^ D10 cells are highly resistant and aggressive in melanoma model [21]. More recently, Kumar et al. have shown that CD133^+^ melanoma specific CSCs maintain long term tumorigenic potential under in vivo condition [15]. Furthermore, stem cell associated markers, nestin and CD133 are highly expressed on circulatory melanoma cells which might represent an index of poor prognosis [22]. Other group has shown that receptor activator of NF-κB (RANK) expressing metastatic melanoma cells co-expressed ABCB5 and CD133 [23]. In accordance with previous reports, Schatton et al. have identified malignant melanoma-initiating cells (MMICs) which are capable of self renewal and differentiation and enriched on the basis of preferential expression of markers such as ABCB5 (a member of ATP-binding cassette) [5]. Moreover, it has also been reported that the amplification of ABCB5 is a predisposing factor for melanoma development which further emphasize the specific role of stem cells in melanoma growth [24]. In addition, ABCB5^+^ melanoma cells showed tumor initiation at the level of 1x10^5^ cells, whereas 100-fold more ABCB5^−^ cells are required to develop tumor under in vivo condition indicating the importance of CSCs in melanoma progression. In addition, PD-1^+^ and B7.2^+^ cells in human melanoma are responsible for higher tumorigenicity compared with PD-1^−^ and B7.2^−^ cells respectively. It has also been observed that the expression of PD-1 and B7.2 markers are coexpressed with ABCB5 [25]. Further, Fang et al. showed that CD20^+^ fraction from melanoma cells exhibit multipotent properties under in vitro and in vivo conditions [3]. The expression of VEGFR1 is highly upregulated and associated with tumor progression in malignant melanoma-initiating cells. In this study, the expression of VEGFR1 and its downstream signaling play a crucial role in ABCB5^+^ MMIC which govern the vasculogenic mimicry (VM) and higher tumor growth [9]. Moreover, Schlaak et al. have showed that elimination of CD20^+^ cells lead to regression of metastatic melanoma [26]. Furthermore, CXCR6 is a newly defined biomarker for identification and characterization of aggressive melanoma specific CSCs [27]. Civenni et al. have also characterized the CSCs based on expression of CD271, a specific melanoma stem cell marker [6]. In contrast, Boyle et al. have demonstrated that CD271 expression is unstable and not consistently linked to tumorigenicity in clinical melanoma patient’s specimens [28]. Additionally, Li et al. have shown that the expression of CD271 is epigenetically regulated through DNA methylation. In this study, they have shown that expression of CD271 induced drastically when treated with 5-aza (an inhibitor of the methylase enzyme) for 6 days demonstrating that DNA methylation is involved in regulation of CD271 expression [29].

Roesch and their colleagues demonstrated that JARID1B^+^ melanoma cells are slow-cycling that are responsible for giving rise to a highly proliferative progeny [30]. In addition, Kumar et al. have shown the existence of side population (SP) in melanoma which exhibits the properties of CSCs [10]. Recently, Luo et al. have well established the concept of CSCs in melanoma based on the intracellular stem cell marker ALDH where they have shown that ALDH^+^ cells fulfil the criteria of self-renewal and differentiation of CSCs upon serial transplantation into NOD/SCID mice. ALDH1A is a super-family of detoxifying enzymes which metabolize a wide variety of intracellular aldehydes therefore providing chemoresistance in human melanoma stem cells, thus governing cancer cell proliferation and survival [7]. Recently, it has been observed that CD44^high^/ALDH1A1^high^ cells were significantly higher in melanoma specimens which suggest a possible candidate for targeted therapy of skin cancer aiming to CSCs [31]. Dioxin receptor (AhR) integrates signaling pathways associated with xenobiotic metabolism and tissue or organ homeostasis. AhR is involved in dualistic role in tumor development. However, AhR knockdown increased ALDH1A1 activity and enhances B16F10 melanoma growth through maintaining cancer stem-like phenotypes. Further, knockdown of ALDH1A1 reduced the levels of CD133^+^/CD29^+^/CD44^+^ cells, melanosphere size and expression of Sox2, a pluripotency factor in AhR knockdown cells [32]. Taken together, these results demonstrate that existence of a subset of cells in melanoma with CSC-like features that can be identified based on specific unique markers.

### The crosstalk between melanoma CSCs and angiogenesis

**Fig. 1:**
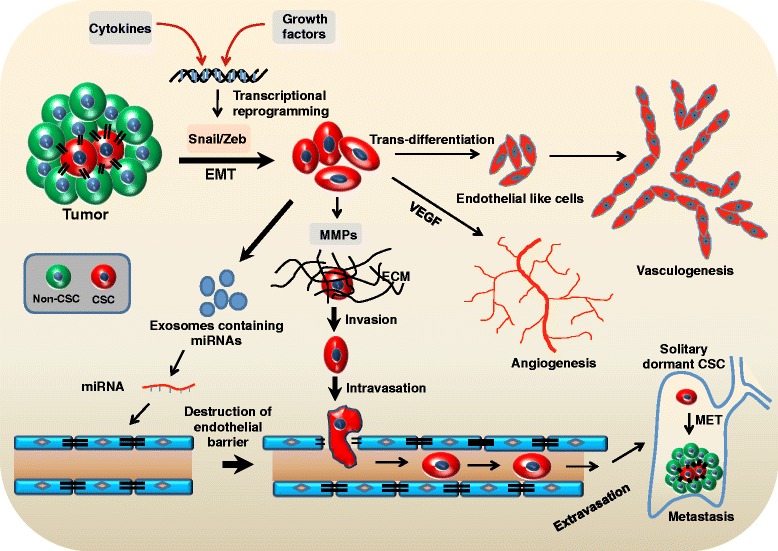
Role of CSCs in melanoma growth, metastasis and angiogenesis. Cytokines and growth factors transcriptionally reprogram the several transcriptional factors that mediate EMT. During EMT, cells lose the epithelial markers and acquired the mesenchymal phenotypes which help in the invasion. CSCs degrade ECM proteins through MMPs which enhance the invasion properties. Further, microenvironment associated factors trans-differentiate CSCs into endothelial-like phenotype that leads to enhance neovascularisation. Additionally, angiogenesis is enhanced through upregulation of CSCs-derived angiogenic factors such as VEGF in melanoma. The CSCs-derived exosomes contain specific miRNA that control the endothelial barriers and promote intravasation that ultimately regulates metastasis. Upon change in physiology of CSC niche, dormant metastatic CSCs reactivate and exhibit MET phenotype leading to establishment of secondary tumors. CSCs: cancer stem cells; EMT: epithelial to mesenchymal transition; MET: mesenchymal to epithelial transition; ECM: extracellular matrix; MMPs: matrix metalloproteinases; VEGF: vascular endothelial growth factor

Angiogenesis is an important hallmark of tumor development. Most of the genes that are upregulated in aggressive melanoma are known to be involved in angiogenesis and vasculogenesis, such as CD144, EPHA2 and LAMC2. These molecules are required for formation and maintenance of blood vessels. Some of these genes are also involved in vasculogenic mimicry leading to melanoma progression and metastasis [33]. Jin et al. have demonstrated the existence of EGFR-Akt-Smad signaling in stem like cells which promotes tumor angiogenesis by ID3 regulated cytokine induction [34]. Neovascularization in tumor is often associated with CSC-derived endothelial cells (ECs). Kumar et al. and Bussolati et al. have shown that renal and melanoma derived-CSCs are able to differentiate into endothelial like cells when cultured in endothelial cell growth specific medium [15, 35]. Cumulative evidences have shown that CSCs are involved in the angiogenesis phenomenon. Monzani et al. have demonstrated that WM115 cells express angiogenic factors like VEGF, VEGFR-2, Ang1/2 and Tie2 along with melanoma specific CSCs signaling such as Notch4 [4]. Since melanoma specific CSCs have high degree of differentiation plasticity, they may contribute to the de novo formation of tumor blood vessels via a process termed vasculogenic mimicry (VM) [33]. In accord with these results, Frank et al. have demonstrated that ABCB5^+^ human melanoma cells are specifically associated with vasculogenic mimicry by expressing endothelial specific and other angiogenic proteins. The same group has shown that human melanoma ABCB5^+^ subpopulation preferentially expresses the vasculogenic differentiation markers like Tie1 and CD144 (VE-cadherin) which are distinct from those expressed on mature CD31^+^ tumor endothelial cells [9]. CD133^+^ melanoma specific CSCs exhibit the functional tube formation and maintain the endothelial cell alignment through secretory factors present in their conditioned medium [15] as shown in Fig. 1. In addition, ABCB5^+^ and CD133^+^ melanoma specific CSCs preferentially express VEGFR1 and VEGF that are essential for VM in human melanoma cells [9, 15].

Lai et al. have identified that CD133^+^ and ABCB5^+^ subpopulations are colocalized in the perivascular niche of melanoma. This perivascular niche contains CD144 (VE-cadherin)^+^ melanoma cells with vessel like channels. They have further investigated the role of CD133^+^ cells in vasculogenic mimicry using CD133^+^/GFP^+^ melanoma cells under in vivo conditions. They have observed that vascular niches containing mosaic vessels that are partially lined up by both CD144^+^/GFP^+^ channels-forming melanoma cells and CD144^+^/GFP^−^ mouse endothelial cells [36]. Schnegg et al. have also demonstrated that perivascular niches exhibit higher accumulation of CD133^+^ and CD271^+^ melanoma stem cells [37]. It has been reported that CSCs from glioblastoma help in tumor vascularisation through recruitment of endothelial progenitor cells (EPCs) via VEGF and SDF1 [38]. Recently, it has been observed that melanoma cells educate mesenchymal stromal cells towards vasculogenic mimicry through various angiogenic factors [39]. Furthermore, a recent study shows that CD133^+^ D10 melanoma cells exhibit a significant induction of early angiogenesis under in vivo condition compared with CD133^−^ D10 cells [40]. Interestingly, VM-forming melanoma cells are positive for CD271 [41] and the data supports that VEGFR1 and PKCα signaling control melanoma VM [42].

Moreover, Harell et al. have showed that primary melanoma induces sentinel lymph node lymphangiogenesis prior to initiation of dissemination [43]. Subsequent study demonstrated that VEGF-A in cutaneous squamous cell carcinoma and VEGF-C in melanoma induces the sentinel lymph node lymphangiogenesis and promotes lymphatic metastasis [44, 45]. However, the detailed mechanism of melanoma specific CSCs in lymphangiogenesis need to be investigated further. All these observations clearly emphasized the role of melanoma specific CSCs in angiogenesis and lymphangiogenesis leading to melanoma growth.

### Melanoma CSCs in metastasis and tumor recurrence

Metastasis is the intermediate phenomenon of the tumor development. Tumor metastasis is established by several phenotypic changes and processes such as invasive growth, escape from primary site, intravasation, lymphatic and haematogenous survival, extravasation and colonization at distant organs. During metastasis, tumor cells lose or gain several adhesion molecules such as ALCAM, VE-cadherin, L1-CAM, integrin β3, ICAM-1, E-cadherin and N-cadherin [46]. Malignant melanoma is a highly metastatic disease having survival period of less than 5 years. It mostly metastasizes to lungs and also affects other visceral organs. Several clinical reports suggest that regional lymph node metastasis is a determinant of outcome for patients with melanoma. The presence of regional lymph node metastasis is commonly used as an indication for systemic and adjuvant therapy. However, the potential risk of recurrence greatly varies in each individual due to heterogeneous nature of the tumors [47].

Al Dhaybi et al. and Rappa et al. have shown the existence of CD133^+^ CSCs in development of malignant melanoma and their potential to metastasize in lymph nodes, lung and/or other visceral organs. These cells expressed low level of proliferative maker Ki-67 that may associate with the chemoresistant ability of CSCs [48, 49]. Fusi et al. have observed the co-expression of CD133 with Nestin on circulatory melanoma cells. These data highlighted that the patient survival rate is low with Nestin overexpressed circulatory melanoma cells compared to low Nestin expressed cells [22]. Additionally, Klein et al. observed significant increase in the expression of stem cell markers, CD133, CD166 and Nestin in primary and metastatic melanoma cells [50]. Furthermore, it has been shown that CD20^+^ CSCs are responsible for metastasis [26]. Moreover, Civenni et al. and de Waard et al. have also established the link between metastasis and CD271^+^ or ABCB5^+^ melanoma stem cells [6, 51]. In addition, Kumar et al. have shown that side population in melanoma cells have higher metastatic capacity as compared with those of non-side population [10]. Several reports suggest that ALDH1 is a potential marker in CSCs derived from melanoma. Genetic ablation of ALDH1A1 by its specific shRNA resulted not only significant reduction in tumor growth but also exhibited significant decrease in metastatic burden in melanoma [52]. Furthermore, clinical data strongly suggests that RANK is highly upregulated on melanoma-initiating cells and the expression of RANK is higher in metastases as compared to primary tumor [23]. Zhao et al. have recently showed that CD133^+^CD44^+^ melanoma CSCs are highly metastatic towards lung [53]. During invasion, tumor cells exhibit different phenotypic changes through epigenetic modifications. Studies revealed that combined expression of EZH2, H3K4me2 and H3K27me3 might correlate with potential CSCs properties. Moreover, the expressions of EZH2, H3K4me2 and H3K27me3 were enhanced significantly at the invasive site of tumor. However, expressions of these molecules were less in metastatic sites as compared to patients with primary melanoma cases [54].

Additionally, recent clinical data showed that there were significant increase in the number of ABCB5^+^CD271^+^RANK^+^ CSCs in circulatory tumor cells (CTCs) at late stage of melanoma. These data indicate that CTCs are highly enriched in CSCs which are responsible for establishment of distant secondary tumors [55]. Ojha et al. have reported that autophagy in CSCs establish a potential link between chemoresistance, metastasis and recurrence in several tumors [56]. Several lines of evidences suggest that some of the metastasized tumor solitary cells exist in a quiescent like state accompanied by decreased expression of proliferation specific markers. The tumor dormancy might caused by several mechanisms such as stress induced by microenvironment, programming of the transcriptional factors and therapeutic treatment of the primary tumor [57]. Additional data also suggest that CSCs are predominantly quiescent in nature and that may contribute to dormancy [58]. Changes in microenvironment including pro-proliferative, pro-inflammatory and pro-angiogenic molecules may lead to the mobilization and activation of dormant CSCs [57]. Stereotactic body radiation therapy promotes recurrence of melanoma through mesenchymal stem cells (MSCs) recruitment and pericytes differentiation leading to vasculogenesis [59]. The exosomes enhance the metastatic behaviour of primary tumors by educating bone marrow progenitors through MET (a receptor tyrosine kinase) from highly metastatic melanoma [60]. Gao et al. have demonstrated the mediator and suppressor molecules for metastatic reactivation in breast cancer cells by using forward genetic screening approach in mice [61]. The immune-surveillance may induces dormancy in single cutaneous malignant melanoma cells by blocking their proliferation cycle [62]. All these results suggest that CSCs are involved in the formation of metastatic lesion and tumor recurrence (Fig. 1).

### Melanoma CSCs and EMT

In the process of metastasis, cells need to disseminate from their primary site where tumor cells lose the epithelial phenotype and gain the mesenchymal status termed as epithelial-to-mesenchymal transition (EMT). Upon reaching to secondary site, these reprogrammed cells exhibit a reversal process designated as mesenchymal-to-epithelial transition (MET) as shown in Fig 1. Mounting evidences have demonstrated that EMT induced by different factors, is associated with tumor aggressiveness and metastasis and these cells share molecular characteristic with CSCs [63]. EMT is driven by several transcription factors (TF) such as Snail, Slug, Twist and Zeb and studies showed that EMT-inducer control the progression of malignant melanoma [63, 64]. Recent studies also showed that silencing of CD133 downregulates Slug and Snail expression [15]. Yao et al. have shown that epithelial splicing regulatory protein 1 (ESRP1) is associated with EMT in addition to Slug, Snail and Zeb in human malignant melanoma. It has been shown that the expressions of epithelial markers were higher in tumors with full-length ESRP1. In contrast, the expression of mesenchymal markers is higher in tumors with low level of ESRP1 [65]. Recent studies have highlighted that EMT-inducer has antagonistic function in melanoma progression. In melanocytes, the expressions of Snail2 and Zeb2 were found to be higher and act as oncosuppressor whereas Twist1 and Zeb1 promote neoplastic transformation of melanocytes and aberrantly reactivate in melanoma [66]. Other studies have shown that Slug regulates Zeb1 expression in melanoma at transcriptional level through binding to the E-boxes of promoter [64]. Guo et al. have showed that BRAF activates long non-coding RNA (BANCR) that induces EMT phenomenon and contribute to cancer cell migration [67].

Several lines of evidences suggest that EMT plays a crucial role in tumor metastasis and recurrence which is tightly linked with the CSCs biology. Accumulative data indicate that CD133^+^ cells exhibit EMT phenotype and maintained stemness properties [68, 69]. In addition, CD133 facilitates EMT through interaction with ERK pathway [68]. The study also revealed that S100A4, a master mediator for EMT maintains tumor-initiating cells (TICs) [70]. Several reports indicate that EMT promotes CSCs phenotype [70, 71]. Mani et al. have demonstrated that overexpression of Twist and Snail enhances EMT in immortalized human mammary epithelial cells that resulted in acquisition of CD44^high^/CD24^low^ expression and higher mammosphere-forming ability [72]. Zeb1 expression is closely associated with maintenance of CD133^+^CD44^+^ CSCs-like properties in B16F10 cells that include colony formation, drug resistance, migration and invasion. Knockdown of Zeb1 leads to inhibition of tumorigenicity and metastasis in CD133^+^CD44^+^ B16F10 specific CSCs. In addition, downregulation of Zeb1 reverse the EMT phenotype of CD133^+^CD44^+^ CSCs. These data suggested that Zeb1 maintained the CSCs properties and EMT phenotype in melanoma cells [73]. Downregulation of Zeb1, Twist1 and Snail1 attenuates the invasive properties of uveal melanoma cells [74]. GLI transcription factor is identified as the effectors of the Hedgehog signaling pathway. Apart from Zeb, Snail and Twist that regulate E-cadherin and GLI-2 forms a complex with Zeb1 and exhibits the repression of E-cadherin in human melanoma cells [75]. It is well documented that microphthalmia-associated transcription factor (MITF) determine the cell fate of melanocyte. In addition, Zeb2 is needed for proper differentiation of melanocyte through regulation of MITF-ZEB1 transcription network. Knocking down of Zeb2 leads to significant downregulation of MITF and concomitant upregulation of Zeb1, Vimentin and Fibronectin resulted in enhanced melanoma progression [76]. Recent study also supports that insulin-like growth factor binding protein 5 (IGFBP5) acts as a tumor suppressor in human melanoma by inhibiting EMT phenotype and attenuates the expression of stem cell markers such as Nanog, Sox2, Oct4, KLF4 and CD133 [77]. In contrast, immune-related GTPase family protein IRGMI induces B16 melanoma cell migration, invasion and EMT through F-actin polymerization [78]. In addition to EMT, the mesenchymal to amoeboid transition (MAT), a second type of motility shift is essential for melanoma tumor progression. The programming of MAT exhibits increased in stem-like and colonogenic features of melanoma cells. Overexpression of EphA2 or RacN17 in melanoma cells induces MAT like phenotype which leads to increase in tumor invasion [79].

Several solid tumors including melanoma exhibit extracellular acidosis. Peppicelli et al. showed that exposing melanoma cells with an acidic extracellular environment (pH 6.7) upregulate the expression of mesenchymal markers such as N-cadherin, Vimentin whereas the expression of epithelial specific marker such as E-cadherin was found to be downregulated. Further, these data also suggest that acidic environment enhanced the melanoma cell invasion and lung colonization through upregulation of MMP-9 activity [80]. Apart from several cytokine and growth factor, TGFβ acts as key player to induce EMT in several cancers including breast and melanoma [72, 81]. In addition, TGFβ also promotes amoeboid feature that leads to higher melanoma migration and dissemination [82]. Overall, these reports indicate that CSC is linked with EMT features in association with several microenvironmental factors (Fig. 1).

### Role of CSCs in regulation of immune cells in melanoma

Compelling evidences suggest that tumors are immunogenic in nature and melanoma is one of the well characterized model [83]. Melanoma cells display multiple antigens and peptide epitopes that help the host immune system to respond either serologically or through cell-mediated mechanisms [83]. However, the question remained unsolved why tumors cannot be eliminated by the immune system. CSCs are responsible for recurrence of tumors and are associated with immune escape mechanism [13]. Therefore, to prevent the contribution of CSCs in tumor growth, several groups have studied whether the effector immune cytotoxic cells like NK cells, CD8 T cells and γδT cells could eliminate the CSCs compartments [84].

Recently, it has been shown that anti-apoptotic proteins such as Bcl2, Bcl-xl or survivin not only protect CSCs against chemotherapeutic agents, but also enhance resistance for apoptosis-inducing immune effectors like NK- or T- cells [13]. The data revealed that due to low level of expression of MHC class I molecule, these CSCs are poorly recognized by T lymphocytes. However, CSCs can be eliminated by γδT lymphocytes upon sensitization with bisphosphonate zoledronate [85]. Recent data demonstrate that CSCs derived from colon cancer, glioblastoma as well as melanoma can be recognized by NK cells [84]. In contrast, Pietra et al. have shown that melanoma cells impair the function of NK cells through inhibiting the expression of major receptors including NKp30, NKp44 and NKG2D which are associated with cytolytic activity. Further, they have observed that this inhibitory effect was primarily mediated by indoleamine 2,3-dioxygenase (IDO) and prostaglandin E2 (PGE2) [86]. Moreover, overexpression of 6 kDa early secreted antigenic target (ESAT-6), a glycosylphosphatidylinositol (GPI)-anchored form and secreted interleukin (IL)-21 in B16F10 CD133^+^CD44^+^ CSCs leads to activation of anti-ESAT-6 and interferon (IFN)-γ correlates with enhanced anti-melanoma efficacy and prolonged survival of melanoma bearing mice [53]. It has been shown that IL-2 and IL-15 activate NK cells and exhibit enhanced cytotoxicity against CSCs derived from melanoma and breast cancer [87, 88]. Moreover, NK cells preferentially eliminate CD24^+^/CD44^+^, CD133^+^ and ALDH^bright^ CSCs in variety of human cancer cell lines through upregulation of NKG2D ligands [89]. Schattan et al. have shown that ABCB5^+^ malignant melanoma initiating cells exhibit lower expression of melanoma associated antigens such as MART-1, ML-IAP, NY-ESO-1 and MAGE-A which might help them in escaping from immune surveillance mechanism. Furthermore, ABCB5^+^ melanoma cells inhibit T-cell activation through IL-2 and induce CD4^+^CD25^+^FoxP3^+^ regulatory T (Treg) cells via B7.2 dependent manner [25]. However, CD271^+^ melanoma cells does not express TYR, MART1 and MAGE antigens which propel them for immune resistance against T cells [90]. Moreover, overexpression of CD271 in melanoma cells suppressed melanoma-specific cytotoxic T lymphocytes (CTLs) under in vitro conditions. Additionally, CTLs-derived IFN-γ induces CD271 expression in melanoma cells that is associated with downregulation of production of melanoma antigens [91]. These data demonstrate a novel mechanism for evasion of anti-tumor immunity. In contrast, CD133^+^ melanoma CSCs express high level of cancer/testis (CT) antigens which make them more susceptible against CD8^+^ T lymphocytes [92]. Recently, a new study showed that CD133^+^ murine melanoma cells express DDX3X antigen which is immunogenic and able to protect melanoma growth in T-cell dependent manner [93]. Further, IL-6 induces melanoma differentiation whereas IL-10 supports the enrichment of undifferentiated melanoma stem like compartment [94]. Immune system also regulates the EMT process which is consistent with earlier findings. Kudo-Suito et al. showed that Snail-induced EMT accelerates cancer metastasis through invasion and induction of immunosuppression by CD4^+^Foxp3^+^ Treg cells [95].

### Signaling mechanism in melanoma specific CSCs

In past, significant progress has been made towards understanding the molecular mechanism of malignant melanoma. Several reports suggest that CSCs are responsible for limited tumor response against conventional treatment due to specific intracellular molecular properties [14]. Thus, delineating the signaling pathways by which CSCs control tumor-protective mechanisms will provide the better understanding of tumor relapse.

Signaling mechanism has been extensively studied in embryonic stem cells for their maintenance or self-renewal and these are common in CSCs. Khalkhali-Ellis et al. demonstrated that Nodal which maintain pluripotency of embryonic stem cells and plasticity of melanoma CSCs, interact with heterodimeric receptors of Activin I and II in embryonic stem cells whereas it binds with TGFβR1 and II in metastatic melanoma [96]. Alcohol consumption causes the risk associated with several human cancers. Recent study showed that ethanol exposure to FEMX-I melanoma cells increase the percentage of CD271^+^ CSCs. Ethanol activates NF-κB by decreasing its p50 homodimers which leads to enhancement of CD271 expression [97]. Melanoma specific CSCs are involved in establishment of metastases that is determined by several signaling cascades. Sonic hedgehog (Shh), Wnt or Notch regulatory signaling pathways modulate the differentiation plasticity and promote self-renewal of stem cells [98]. Several reports have shown the presence of these signaling pathways and their therapeutic targets in various types of stem cells. Geng et al. have shown that Hedgehog (HH) pathway play a vital role in development of melanogenesis in murine melanoma models [99]. Moreover, abolishment of HH-GLI signaling pathway drastically attenuates the self-renewal and tumor initiating potential of ALDH^bright^ melanoma CSCs [100]. In addition, Pandolfi et al. have shown that effectors of HH signaling, GLI1/2 regulates transcription factor E2F1 which is essential for cell proliferation and tumor progression in melanoma. Further, E2F1 modulates iASPP (inhibitor of apoptosis-stimulating protein of p53) by directly binding to the promoter region of iASPP and enhance the proliferation indicating that HH-GLI-E2F1-iASPP axis is essential for melanoma progression [101]. Moreover, Wnt signaling has been implicated in regulation of self-renewal and proliferation of normal stem and cancer cells [98]. High level of Wnt receptor, FZD7 is associated with enhanced metastatic potential of melanoma cells. Knocking down of FZD7 suppressed JNK activation and metastatic growth in melanoma [102]. Similarly, Notch signaling plays a critical role in regulating cell to cell communication during embryogenesis, cellular proliferation, differentiation and apoptosis [103]. Notch receptors cleaved by γ-secretase and TACE (Tumor Necrosis Factor-α-Converting Enzyme) resulted in release of NICD (Notch Intracellular Domain) that translocate into nucleus and regulate the promoter activity of various genes. Attenuation of γ-secretase and TACE leads to downregulation of NICD2 and Hes1 which preferentially inhibit melanosphere formation indicating that Notch2 regulates melanoma progression in CSCs [104]. It has been also shown that Notch4 promotes invasion and metastasis in melanoma stem-like cells [105]. Additionally, Akt regulates nucleocytoplasmic shuttling of NICD4 [106]. In addition, activated Notch1 increases the stability of β-catenin that play important role in melanoma cell migration and proliferation [107]. Recent data showed that Notch1 signaling is highly augmented in CD133^+^ CSCs in melanoma. NICD1 binds to the promoter region of CD133 and transcriptionally regulates its expression. Further, Notch1-CD133 signaling axis activates p38-MAPK pathway that leads to AP-1-DNA binding and regulates the expression of MMPs and VEGF that are essential for metastasis and angiogenesis [15]. Moreover, recent study showed that tetraspanin, TM4SF promotes CSCs phenotype in breast cancer cells. Mechanistically, collagen I but not IV, fibronectin and laminin 1 induces TM4SF-mediated coupling of DDR1 to PKCα and augment JAK2-STAT3 signaling which is essential for reactivation of the dormant solitary tumor cells for establishment of multi-organ site metastatic out growth [108].

**Fig. 2:**
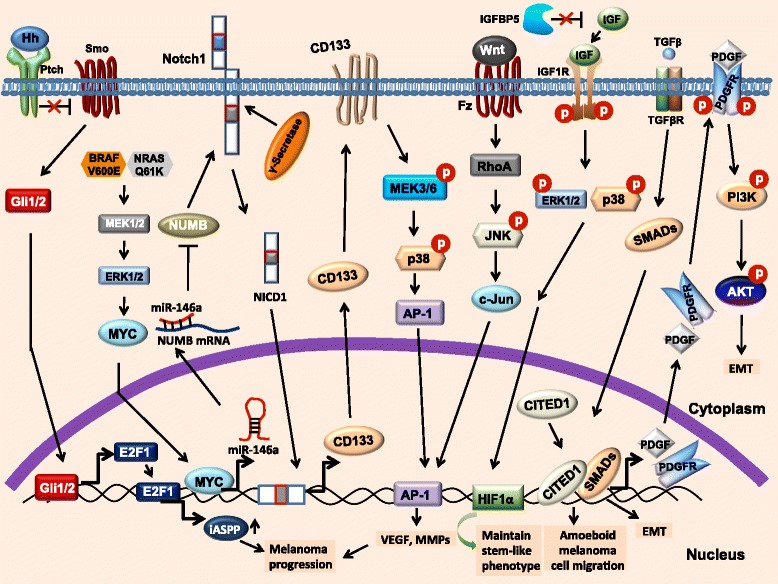
Signaling involved in CSCs that mediates melanoma progression. Hedgehog signaling transcriptionally regulates E2F1 expression and promotes iASPP expression leading to melanoma progression. BRAF (V600E) and NRAS (Q61K) signaling induce miR-146a through MYC. This miRNA enhances the Notch signaling through downregulation of NUMB expression. Notch1 Intracellular Cleaved Domain (NICD1) translocates into the nucleus and transcriptionally regulates the expression of CD133 that preferentially activates the p-38 MAPK pathway-mediated AP-1-DNA binding. Additionally, AP-1-DNA binding also induced by Wnt signaling leading to higher melanoma metastasis and angiogenesis. IGF binds to their receptor (IGF1R) and activates the phosphorylation of ERK and p38 that leads to HIF1α-DNA binding and maintain melanoma stem-like phenotype. Selected EMT modulator such as TGFβ activates PI3K-Akt signaling pathway and induces amoeboidal cell migration and EMT phenotype in melanoma. iASPP: inhibitor of apoptosis-stimulating protein of p53; NICD1: notch1 intracellular cleaved domain; MAPK: mitogen-activated protein kinases; IGF1R: insulin growth factor receptor 1; IGFBP5: insulin growth factor binding protein 5; HIF: hypoxia-inducible factor; TGFβ: transforming growth factor beta; EMT: epithelial to mesenchymal transition

The comparative analysis of monolayer vs 3D spheroid showed that neural progenitor genes that includes ID4 (Inhibitor of DNA Binding 4) switched from 3D-spheroid to highly differentiated morphology indicating that ID4 plays a crucial role in the maintenance of CSCs phenotype in melanoma cells [109]. In contrast, IGFBP5 acts as tumor suppressor in melanoma through attenuation of stem-like cell activity (Fig. 2). IGFBP5 disrupt the binding of IGF to IGFR1 leading to inactivation of ERK1/2 and p38 MAPK pathway that preferentially attenuates HIF1α regulated VEGF and MMPs gene expression [77]. Interestingly, TGFβ markedly induces EMT phenotype in several cancers including breast and melanoma. Schlegel et al. have demonstrated that PI3K and PDGF signaling are important for TGFβ-induced EMT in human melanoma cells. TGFβ activates SMAD signaling which in turn regulates PDGF and its receptor expression leading to activation of PI3K-Akt pathway which contributes EMT in melanoma [81]. TGFβ also induces the amoeboid migration of melanoma cells which is alternative of EMT. Downstream of TGFβ, SMAD2 and its adaptor CITED1 regulate the amoeboidal characteristic of melanoma cells. Moreover, TGFβ-SMAD2-CITED1 signaling axis induces melanoma cell attachment to endothelial cells, lung colonization and metastatic out growth [82]. Rh123^low^ (Low Rhodamine 123) cells exhibit stem-like phenotype correlate with enhanced levels of HIF1α, Oct4 and ABCB5 and reduced level of Cyclin D1 and CDK4 which define the quiescent and chemoresistance properties of CSCs in melanoma. It has been also reported that PI3K/Akt pathway is involved in the maintenance of Rh123^low^ in melanoma stem cell compartment [110]. Attenuation of PI3K/Akt downregulates TNF-mediated enrichment of GFP^high^ label-retaining in CSCs in melanoma [111]. Vasculogenic mimicry (VM) plays a crucial role in melanoma angiogenesis. VEGF-A is a well known regulator of tumor vascularisation. Inhibition of VEGFR2 kinase activity with PTKi-II (protein tyrosine kinase inhibitor II) does not affect VM in melanoma cells however, attenuation of VEGFR1 significantly disrupts this process. In addition, inhibition of PKCα abrogates vasculogenic mimicry. Taken together, these data suggest that VEGFR1 and PKCα signaling regulates VM in melanoma [42]. In addition, deficiency of p63, a p53 homolog suppresses tumor growth. Interestingly, the isoform of p63, ∆Np63α bypasses the senescence to promote stem-like cell proliferation and tumorigenesis in skin cancer under in vivo condition [112]. Moreover, ∆Np63α enhances the expression and activation of Akt1 and p-Akt1 which preferentially induces the proliferation and survival of cancer cell [113]. Overall, all these data suggest that molecular signaling plays an important role in CSCs-mediated melanoma progression (Fig. 2).

### CSCs and microenvironment in melanoma

Like normal stem cells, CSCs are regulated by cell to cell communications with their non-tumorigenic cancer population or stromal cells to sustain their own inter-discipline [114]. Tumor progression also depends on several secreted factors or other niche component, site of injection and species-species host milieu. Rao et al. have demonstrated that tumor associated macrophage (TAM) interacts with CSCs and secrete osteopontin (OPN) which enhances the tumorigenicity and clonogenicity in colorectal cancer [115]. These CSCs might also reciprocally regulate surrounding niche through the secretion of specific growth factor that regulates OPN expression in TAM. Moreover, Kale et al. have shown that macrophage in association with melanoma enhances the OPN expression that further regulates Cox2 production and controls melanoma growth and angiogenesis [116]. Kumar et al. have also demonstrated that stromal OPN enriches SP-phenotype which ultimately control melanoma progression [10]. Hypoxic microenvironment plays an important role in tumor progression and controls tumor stem cell population by stabilizing hypoxia inducible factor (HIF) [117]. HIF1 and HIF2 are the subfamily of hypoxia inducible transcription factors which are activated in tumor hypoxic regions and are responsible to initiate a complex set of cellular response in tumor cells. It has been reported that HIF1α and HIF2α are involved in invasion and invadopodia formation and associated with melanoma metastases in patients. HIF1α increases VEGF expression and decreases E-cadherin levels that are crucial for angiogenesis and metastasis [118, 119]. Furthermore, hypoxic microenvironment enriches the higher expression of CD133 and VEGFR2 as compared with normoxic conditions that lead to enhanced melanoma growth [120]. Additionally, MFG-E8 induces VEGF and ET-1 expression in MSCs and enhances M2 macrophage polarization that leads to higher angiogenesis and melanoma growth under hypoxic conditions [121]. Taken together, these studies established the role of tumor microenvironment in CSCs-mediated melanoma progression.

### Function of miRNA in melanoma specific CSCs

Several reports have suggested that miRNAs may act as early diagnostic and prognostic biomarker in many cancers including melanoma [122]. Initially, miRNAs that suppress or promote metastasis have been identified in breast cancer [123, 124]. Subsequent studies have revealed that many miRNAs regulate tumor growth, angiogenesis and metastasis in various other cancers. Additionally, the expression of miRNA strongly correlates with various steps of melanoma progression (Fig. 1 and 2). Therefore, deregulation in miRNA expression and function appears to be a pervasive feature of human cancers (Table 2).

**Table 2:** miRNAs associated with CSCs in melanoma

miRNA	Functions	References
miR-10b, miR-21, miR-200c, miR-373 and miR-520c	Regulate EMT	[16]
miR-340-5p	Controls ABCB5 expression	[128]
miR-200c	Regulates Zeb1 expression, cell proliferation and invasion	[129]
miR-33b	Suppresses EMT and cell migration	[130]
miR-885-5p	Maintains stemness feature and controls proliferation and metastasis	[134]
miR-9	Decreases proliferation and metastasis	[135]
miR-155	Regulates NK cell activity	[158]
miR-34a	Target Notch1 signaling pathway	[159]
miR-222	Controls Wnt and PI3K-Akt pathways and regulates CSCs phenotype	[160]

Several studies have shown that cluster of miRNA such as miR-1908, miR-199a-3p and miR-199a-5p drive metastatic invasion, endothelial recruitment and angiogenesis. Further, these miRNA cooperatively attenuates ApoE and DNAJA4 which are required for suppression of cell invasion and endothelial recruitment by engaging LRP1 and LRP8 that ultimately associates with metastatic progression [125]. miRNA profiling revealed that miR-125a-5p suppresses melanoma growth through down regulation of TGFβ signaling by direct targeting Lin28B, a well known inhibitor of Let-7 miRNA biogenesis. Further, clinical data indicated that Lin28B was aberrantly expressed in large number of melanoma patients [126]. A direct plasma assay has been developed to detect circulating miRNA-210 as indicator which can be used for early metastatic recurrence in melanoma under hypoxic environment [122]. In depth studies also revealed that there are differential expression patterns of miRs which correlate CSCs and EMT phenotypes. These data suggest that metastasis and EMT associated miR-10b, miR-21, miR-200c, miR-373 and miR-520c are highly upregulated in melanosphere as compared with monolayer [16]. Tumor derived exosomes also contain miRNAs including miR-105 which help in destroying the vascular endothelial barrier [17].

Noman et al. have shown that hypoxia-inducible miR-210 regulates the susceptibility of tumor cells against cytotoxic T cells [127]. They have shown that hypoxia predominantly induces miR-210 expression in melanoma cells through HIF1α-dependent manner. Further, miR-210 confers resistant in hypoxic tumor cells against cytotoxic T cell-mediated lysis through targeting PTPN1, HOXA1 and TP53I11 genes. The results speculate that this miRNA must have role in immune suppression in hypoxic regions of melanoma where CSCs and metastatic phenotypes are known to evolve [127]. In contrast, other groups have shown that hypoxia induces downregulation of miR-340-5p expression which is responsible for upregulation of melanoma-stem cell associated marker, ABCB5 [128]. Additionally, overexpression of miR-200c in CD44^+^CD133^+^ CSCs resulted in downregulation of Zeb1 expression, reduction in cell proliferation, colony formation, cell migration and invasion as well as tumorigenic potential in melanoma [129]. Moreover, miR-33b suppresses EMT and migratory potential of melanoma cell by direct binding to 3’-UTR of HMGA2 and suppresses its expression [130]. miRNA also help in the communication between cancer cell and their microenvironment. The co-culture of melanoma cells with astrocytes downregulates miR-768-3p expression in melanoma cells which confer the chemoresistance and CSCs properties [131]. Moreover, several other miRNAs also act as mediator and communicators with tumor-associated macrophage (TAM), cancer-associated fibroblast (CAF), cancer-associated endothelial cell (CAEC) and cancer-associated mesothelial cell (CAMC) [132]. Most of the melanoma develops due to BRAF and NRAS mutation. miR-146a is regulated by BRAF and NRAS genes as shown by small RNA profiling. Further, BRAF-MEK-ERK signaling enhances the expression of miR-146a through transcriptional regulation and protein stability. Overexpression of miR-146a increases human melanoma cell proliferation and promotes tumor initiation by targeting NUMB mRNA, a repressor of Notch signaling. A single nucleotide C to G somatic mutation in miR-146a causes enhanced Notch signaling and promotes oncogenesis [133]. Oncogenic DNp73, a dominant-negative variant of tumor-suppressor p73 confers enhanced stem-like properties in melanoma through attenuation of miR-885-5p which regulates IGF1R that is responsible for expression of stemness marker [134]. Augmentation of miR-9 significantly decreases melanoma cell proliferation and migration. This miRNA attenuates the expression of Snail1 with concomitant increase in E-cadherin expression. Mechanistically, miR-9 binds to 3’-UTR of NF-κB and attenuates their expression which preferentially inhibits Snail1 that ultimately leads to inhibition of melanoma cell proliferation and metastasis [135].

### Therapeutic implications of melanoma specific CSCs

Cancer stem cells rarely divide and have distinct cellular physiology from the remaining bulk of tumor population. Traditional chemotherapy and radiation therapy are not sufficient to eradicate these CSCs from patients with cancer. Since CSCs have high level of transporter that pump out chemotherapeutic agents which make CSCs more chemoresistant. CSCs are also radio-resistant because of preferential activation of DNA damage checkpoint and DNA repair capacity [136]. In order to control melanoma growth, it is necessary to target melanoma stem cells because it governs the recurrence of tumors and metastasis after many years and may act as reservoir of therapeutically resistant cells.

**Fig. 3:**
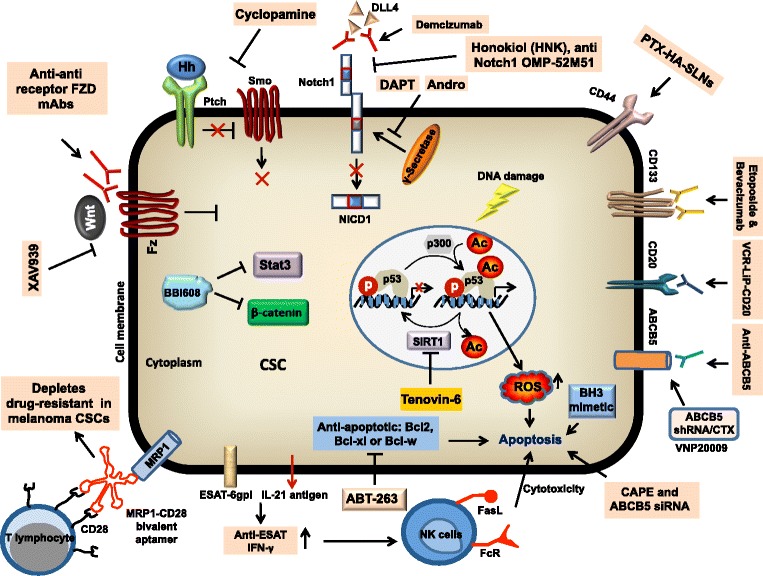
Molecular targeted therapy in melanoma CSCs. mAbs against FZD receptor, DLL4, Notch1, CD133, CD20 or ABCB5 attenuates the CSCs-dependent melanoma progression. Abrogation of Notch1, Hedgehog and Wnt siganling using DAPT, Andro, Honokiol, Cyclopamine or XAV939 depletes CSCs in melanoma. Anti-apoptotic small molecule inhibitors such as ABT-263 and a SIRT1 inhibitor, Tenovin-6 induce apoptosis and suppress CSC-mediated melanoma growth. Overexpression of ESAT-6gpi/IL-21 antigen sensitizes CSCs against NK cell-mediated apoptosis. MRP1-CD28 bivalent aptamers attenuate drug-resistance of CSCs in melanoma. CSCs: cancer stem cells; Hh: hedgehog; Fz: frizzled; DAPT: N-[N-(3,5-Difluorophenacetyl)-L-alanyl]-S-phenylglycine t-butyl ester, a γ-secretase inhibitor; SIRT1: sirtuin 1; ESAT-6gpi: 6 kDa early secreted antigenic target (ESAT-6) in the glycosylphosphatidylinositol (GPI)-anchored form; IL-21: interleukin-21; NK cells: natural killer cells; MRP1: multidrug resistant proteins 1; CTX: cyclophosphomide

Melanoma specific CSCs carry specific marker (CD133, CD20, ABCB5, CD271 and ALDH1) or antigens, so targeting these cells using monoclonal antibodies could help to combat melanoma growth. Rappa et al. have demonstrated that downregulation of CD133 in human metastatic melanoma cells (FEMX-1) attenuates the melanosphere formation and metastatic potential. Further, monoclonal antibodies against different epitopes of CD133 showed dose-dependent cytotoxic effect [49]. Since melanoma specific CSCs express CD20, therefore rituximab therapy is used in clinical trials to treat metastatic melanoma patients by targeting CD20^+^ cells. The CD20 antibody therapy depletes CD20 positive melanoma cells and eliminates peripheral B cells that elevates in malignant melanoma patients [26]. Vincristine (VCR) is commonly used for melanoma therapy however it is ineffective against melanoma specific CSCs. Song et al. investigated that VCR-containing immuno-liposome conjugated with CD20 antibody (VCR-Lip-CD20) is 1.85 fold more effective than VCR alone in melanoma. They have further demonstrated that VCR-Lip-CD20 selectively eliminates CD20^+^ melanoma cells and attenuates tumorigenic ability of WM266-4 melanosphere under in vivo condition [137]. Etoposide alone is not able to eliminate CD133^+^ melanoma specific CSCs that express high level of VEGFR2. However, combination of Etoposide with Bevacizumab significantly induces apoptosis and abolishes sphere-forming ability of CD133^+^ CSCs in melanoma [120]. Schatton et al. have also demonstrated that there was selective elimination of ABCB5 population in melanoma using a monoclonal antibody against ABCB5 under in vivo mice model [5]. Recently, ABCB5^+^ cells in melanoma have shown to suppress T cell activation and thus have specific role for immune evasion [5]. Therefore, targeting the immune system in melanoma patient with IL-2 and IFN-α could be important therapeutic approach [138]. Similarly, Biasco et al. and Flaherty et al. have shown that Temozolomide and Dacarbazine (DTIC) could be important therapeutic agents for treatment of metastatic melanoma [139, 140]. Since CSCs are also maintained by specific signaling cascade therefore targeting these cells using DAPT (Notch inhibitor), Cyclopamine (Hh signaling inhibitor), XAV939 (Wnt signaling inhibitor), or DTIC could be appropriate strategies for treatment of melanoma patient [141–143]. In addition, Demcizumab (anti-Notch ligand, DLL4 antibody), OMP-52M51 (anti-Notch1 antibody), OMP-18R5 (anti-Wnt receptor, FZD monoclonal antibody) and BBI608 (inhibitor of Stat3 and β-catenin pathways) could be better therapeutic agents to combat melanoma [144] (Fig. 3). Recent studies have shown that Andrographolide (Andro), derived from *Andrographis paniculata*, attenuates tumor growth through abrogation of Notch1-mediated CD133-dependent p38 MAPK activation pathway in CD133^+^ melanoma cells. In addition, Andro also impairs the EMT, angiogenesis and metastasis properties of these CD133^+^ cells. Similar to DTIC, Dabrafenib or Trametinib those commonly used for treatment of melanoma, Andro also targets CD133^+^ CSCs and suppressed melanoma growth and lung metastasis [15]. These data indicated that Andro may act as a potential anti-cancer agent for the eradication of CSCs-dependent melanoma progression.

Recent advancements in targeting Bcl2 family members are an alternative option to combat melanoma and overcome relapse or resistance of melanoma. To prevent this relapse, it is necessary to develop effective therapies that eradicate all subpopulation of tumor cells including resistant CSCs subpopulation in melanoma. Bcl2 family members play a crucial role in cancer resistance mechanism and contributes to chemoresistant ability of CSCs and their survival [145, 146]. Several Bcl2 proteins are downstream of commonly activated RAS/BRAF/MAPK and PI3K/Akt signaling pathways which play an important role in tumor initiation and maintenance of melanoma specific CSCs compartment [146]. Small molecule inhibitor such as BH3 mimetic which mimics the pro-apoptotic BH3 protein and induces apoptosis is currently a successful approach. Additionally, ABT-263 is a mimetic of BAD that inhibits anti-apoptotic proteins such as Bcl2, Bcl-xl and Bcl-w [145, 146]. Another, small molecule ABT-737 that inhibits Bcl2, Bcl-xl or Bcl-w is a promising agent for treatment of cancers. Moreover, synthetic retinoid fenretinide N-(4-hydroxyphenyl) retinamide (4-HPR) is another promising agent for the management of breast cancer. Interestingly, the data shown that combination of ABT-737 and 4-HPR significantly eliminate ALDH^+^ CSCs in multiple melanoma cell lines including BRAF and NRAS mutant cells [145]. Further, combination of anti-apoptotic MCL-1 protein inhibitor, SC-2001 and ABT-737 significantly depletes ALDH^+^ cells in melanoma [147]. Most of the CSCs exhibit chemoresistance through attribution of enhanced drug efflux mediated by ATP-binding cassette sub family B (ABCB). The results revealed that caffeic acid phenethyl ester (CAPE), a bioactive molecule induces apoptosis in ABCB5 knocked down CD133^+^ chemoresistant melanoma cells. CAPE activates E2F1 gene which trigger apoptosis through mitochondrial dysfunction, ER stress and induction of pro-apoptotic genes such as Bax, Noxa and Puma. These observations suggest that combination of ABCB5 siRNA and CAPE can de-bulk the tumor mass and eliminate chemoresistance in melanoma specific CSCs [148]. Recent study also demonstrates that inhibition of deacytylase activity of Sirtuin 1 and 2 (SIRT1/2) by Tenovin-6 induces apoptosis in uveal melanoma by upregulating the expression of tumor suppressor gene, p53 and elevation of ROS. Tenovin-6 eliminates the ALDH^+^ CSCs compartments and inhibits the growth and migration of uveal melanoma [149].

Despite recent advances in immunotherapy for cancer, the efficacy of this strategy remains limited. Several studies have indicated that CSCs are weak in immunogenicity due to low expression of antigens which is one of the prime obstacles for induction of anti-tumor immune response. Overexpression of ESAT-6-gpi and IL-21 antigens in CD133^+^CD44^+^ melanoma specific CSCs enhanced the levels of anti-ESAT-6 and interferon (IFN)-γ as well as increased cytotoxic activities of NK cells, splenocytes and complement dependent cytotoxicity leading to attenuation of melanoma growth and metastases [53] (Fig. 3). Moreover, ALDH^high^ melanoma CSCs lyaste-pulsed dendritic cells (DCs) act as a better vaccine which leads to significant reduction in tumor growth and lung metastases. Further, administration of ALDH^high^ CSCs-DC vaccine significantly attenuates ALDH^high^ CSCs percentage in primary tumors through specific binding of IgG produced by primed B cell resulting in lysis of target cells in the presence of complement [150]. Recent study revealed a novel bi-specific aptamer which have two CD28 motifs and able to costimulate T lymphocytes and promotes tumor immunity. Other MRP1 motif that is capable to bind MRP1 aptatope of chemoresistant CSCs. The in vivo results revealed that systemic administration of MRP1-CD28 bi-valent aptamer exhibits higher concentration in B16-MRP^high^ tumor as compared with B16 parental tumors which leads to downregulation of B16-MRP^high^ tumor growth efficiently [151].

Several studies have indicated that CD44 is a CSC marker in various cancers including melanoma. It binds specially to hyaluronic acid. Shen et al. have demonstrated that coating solid lipid nanoparticles with hyaluronan (HA-SLNs) allowed targeted delivery of Paclitaxel (PTX) to CD44^+^ B16F10 melanoma cells. PTX loaded HA-SLNs significantly abrogate tumor growth and lung metastasis [152]. Additionally, combined treatment with engineered VNP20009, carrying shABCB5 and Cyclophosphomide (CTX) drastically reduced ABCB5^+^ CSCs that leads to attenuation of melanoma tumor growth and enhanced survival time [153]. Blocking Hedgehog-GLI signaling with smoothened (SMO) and GLI antagonist by Cyclopamine and Gant61 remarkably attenuates tumor initiating properties of ALDH^high^ melanoma stem cells [100]. Moreover, Honokiol (HNK), a biphenolic natural compound reduces the expression of various stem cell markers such as CD271, CD166, JARID1B and ABCB5 in melanoma. Furthermore, HNK also significantly attenuates CSC properties through inhibition of Notch signaling [104]. Therefore, understanding the signaling cross-talk, tumor microenvironment and identification of novel targets in CSCs may allow us for more effective combinatorial antitumor therapies (Fig. 3).

### Limitation, barriers and controversy in melanoma specific CSCs

It has long been recognized that tumors are heterogeneous in nature that is being confirmed with several functional and phenotypic properties to validate the existence of CSCs in many cancer including melanoma. After several decades, debates still continues whether melanoma contain CSCs and the origin of melanoma CSCs. The identification and characterization of CSCs may help in the elimination of CSCs in melanoma. However, CSCs have several limitations such as they exihibit normal stem cell like self-renewal property, DNA repair mechanism, oxidative state and resistance to xenobiotic toxins. Therefore, targeting CSCs in tumor might also affect normal stem cells and hence distinct molecular features of CSCs need to be established for the management of CSC-mediated therapy in melanoma [154].

Several lines of evidences indicate that CSCs present in the melanoma which in turn responsible for diseases progression [3–6]. In contrast, Quintana et al. have extensively demonstrated that phenotypic heterogeneity among tumorigenic melanoma cells is reversible and not hierarchically organized [155]. Additionally, other study showed that only rare human melanoma cells (0.1-0.0001%) are able to induce tumor upon transplantation into NOD/SCID mice model. However, using a highly immunocompromised NOD/SCID interleukin-2 receptor gamma chain null (Il2rg^−/−^) mice model demonstrate that approximately 25% of unselected melanoma cells formed tumor [156]. These results indicate that melanoma does not follow CSCs model and the percentage of tumorigenic cells is common that depends on tumor microenvironment. Moreover, Boiko et al. have showed that CD271^+^ melanoma cells exhibit CSCs properties that depend on immunocompromised mouse strain, site of injection and cell suspension preparation [90]. These observation suggested that the tumorigenic potential of CSCs also depends on the arrival of the fresh tissue from surgical theatre, process of obtaining a single cells suspension to derive highest number of viable cells, exclusion of dead cells and debris, flow cytometry sorting of CSCs with high yield and purity and transplantation time into recipient model after sorting. The intrinsic tumorigenic potential of human melanoma CSCs also defined by microenvironment in immunocompromised mice model. Therefore, it is critical to assess the relevance of CSCs hypothesis in melanoma using appropriate model system [157]. In addition, we have recently demonstrated that melanoma are heterogeneous in nature. CD133^+^ CSCs derived from melanoma cells exhibit long term tumorigenic potential in isograft mice model which partly exclude the possibility of the artificial milieu [15]. These data demonstrate that functional studies are required to identify and characterize CSCs population. Additional studies are needed in order to understand the pathophysiological function of CSCs in tumor progression.

## Conclusions and future directions

Malignant melanoma is a deadly disease with historically poor prognosis. Due to presence of heterogeneous subpopulation and existence of CSCs, it is difficult to completely cure such a devastating disease. The continued attempt for the identification of CSCs in melanoma and other cancers led to promise the field of CSCs research in order to understand the management of cancers. CSCs exhibit extensive contribution in tumor growth, angiogenesis and reactivation in metastatic out growth through several genetic and epigenetic changes, EMT or stromal microenvironmental factors. CSCs are also responsible for therapeutic resistant that led to tumor relapse. Specific signaling mechanisms are required for the maintenance of CSCs in tumor that can maintain their microenvironment. Therefore, CSCs are becoming priority targets for the development of novel antitumor therapy.

The tumor milieu is a critical regulator of melanoma specific CSCs-driven angiogenesis and metastasis. Signaling effectors from ECM or stromal cells can act as EMT or MET inducer or may regulate dormancy at metastatic sites in CSCs. Furthermore, defined cellular programmes allow CSCs to modify their milieu through the autocrine/paracrine signals that increases invasiveness, metastasis and angiogenesis. These programmes also promote CSCs features and associate with the determination of the CSCs fate. Several reports suggest that CSCs are more chemoresistant which show higher expression of drug efflux pump and inhibitor of pro-apoptotic molecules. CSCs are weak in immunogenic nature due to lack of expression of sufficient antigens. Overexpression of specific antigens lead to eradicate melanoma specific CSCs and attenuates tumor progression. Several microRNAs such as miR-200c and miR-33b act as suppressor through targeting specific signaling cascade. It is known that miRNAs can interact with many important regulatory pathways during CSC-dependent melanoma progression such as MAPK/ERK and PI3K/Akt. Therefore, identification of potential therapeutic agents that may regulate these specific miRNAs which will allow to eradicate the root cause of melanoma development, angiogenesis and metastasis. Considering the unique biology of CSCs, there is great need to develop novel and promising approaches for CSCs targeted cancer therapy. Several studies indicate that controversy of melanoma CSCs arises due to inappropriate mice model and lack of proper functional assays. Considering the controversy, limitation and barriers targeting CSCs, future direction of research are needed to establish or identify the distinct features of CSCs with compared to normal stem cells. In this review, we have discussed that there are several small molecule inhibitors (HNK, ABT-737, ABT-263), nanoparticles conjugated drugs (HA-SLNs-PTX), signaling antagonist (Cyclopamine, Gant61), monoclonal receptor antibodies (anti-CD20, anti-CD133, anti-ABCB5) and microRNAs (miR-200c, miR-33b) those could be used as novel therapeutic strategies for management of melanoma. In addition, recent data showed that Andrographolide may act as a potent anti-cancer agent by targeting Notch1 pathway in CSCs that ultimately suppresses malignant melanoma growth (Fig. 3). Moreover, additional studies are required to target root cause of melanoma growth and metastasis using personalized and combination therapy.

## Abbreviations

CSC: Cancer stem cells
DAPT: N-[N-(3,5-Difluorophenacetyl)-L-alanyl]-S-phenylglycine t-butyl ester, a γ-secretase inhibitor
ECM: Extracellular matrix
EMT: Epithelial to mesenchymal transition
ESAT-6gpi: 6 kDa early secreted antigenic target (ESAT-6) in the glycosylphosphatidylinositol (GPI)-anchored form
Fz: Frizzled
Hh: Hedgehog
HIF: Hypoxia-inducible factor
iASPP: Inhibitor of apoptosis-stimulating protein of p53
IGF1R: Insulin growth factor receptor 1
IGFBP5: Insulin growth factor binding protein 5
IL-21: Interleukin-21
MAPK: Mitogen-activated protein kinases
MET: Mesenchymal to epithelial transition
MMPs: Matrix metalloproteinases
MRP1: Multidrug resistant proteins 1
NICD1: Notch1 intracellular cleaved domain
NK cells: Natural killer cells
SIRT1: Sirtuin 1
TGFβ: Transforming growth factor beta
VEGF: Vascular endothelial growth factor
